# P-1252. Comparison of Vancomycin Dose for Trough and Area Under the Concentration – Time Curve Methods

**DOI:** 10.1093/ofid/ofaf695.1443

**Published:** 2026-01-11

**Authors:** Christina G Rivera (O'Connor), Ryan W W Stevens, Kristin Cole, Thomas J Dilworth, Lee P Skrupky

**Affiliations:** Mayo Clinic, Rochester, MN; Mayo Clinic, Rochester, MN; Mayo Clinic, Rochester, MN; AdvocateAuroraHealth, Aurora St. Luke’s Medical Center, milwaukee, Wisconsin; University of Wisconsin Health, Rochester, Minnesota

## Abstract

**Background:**

According to consensus guidelines, area under the concentration–time curve (AUC) is recommended for vancomycin dosing rather than trough-based monitoring, but transition to AUC-based dosing requires substantial staff education and resource allocation. We sought to evaluate the level of congruency between vancomycin total daily doses (TDD) determined using a trough concentration goal of 10-15 mcg/mL compared with an online Bayesian calculator targeting an AUC of 400-600 mg·h/L.

**Figure:**
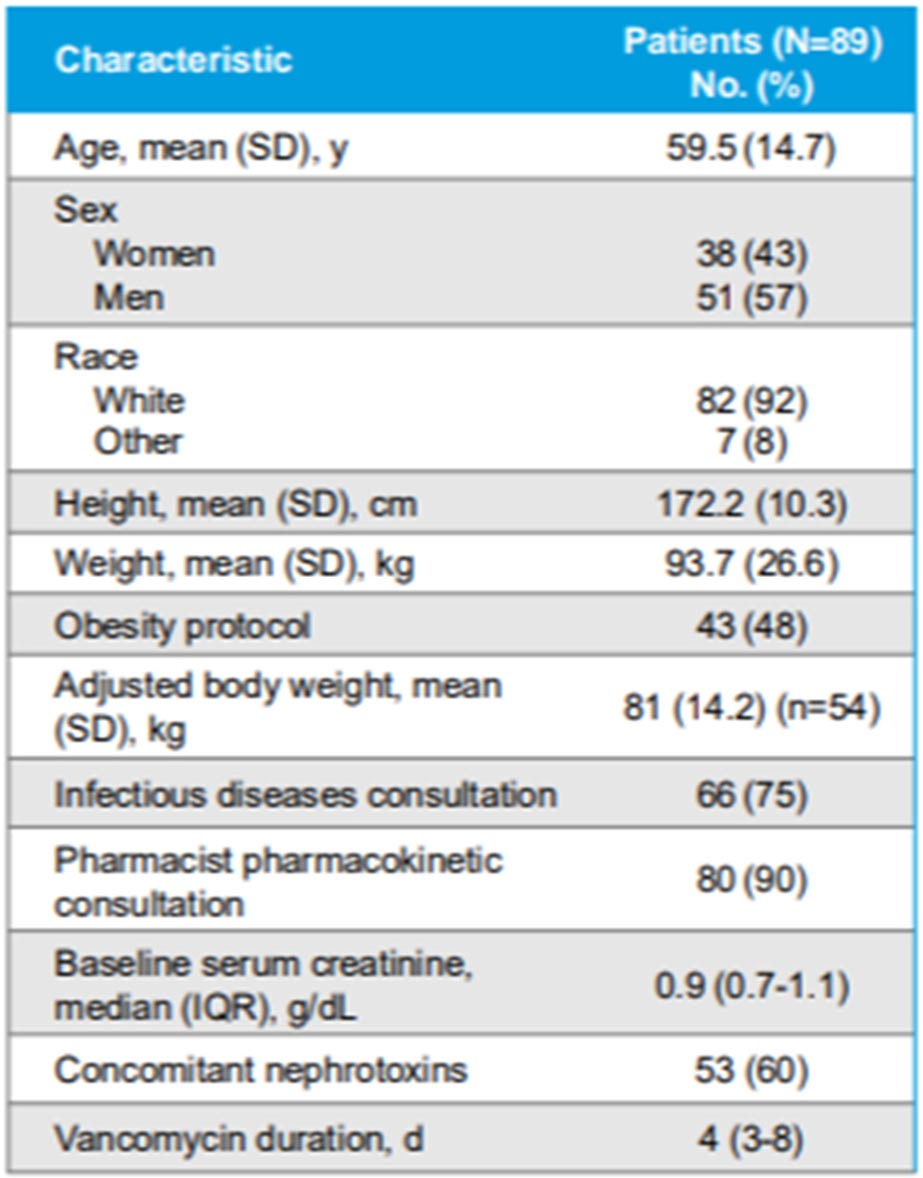
Patient Demographics

**Figure:**
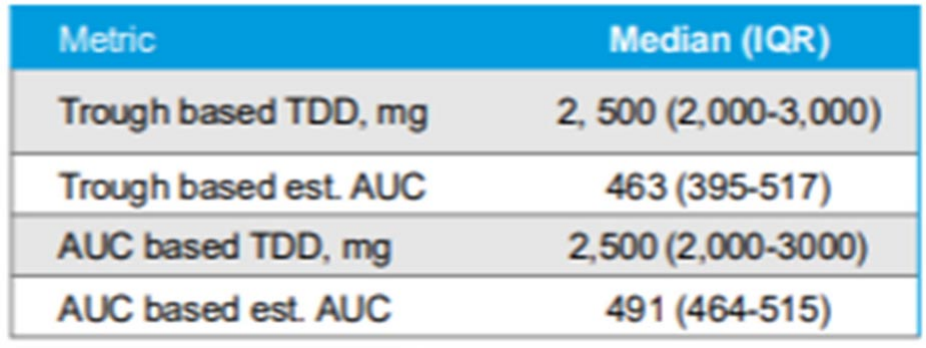
Initial Dose Results

**Methods:**

We performed a retrospective, multicenter cohort study of vancomycin therapeutic drug monitoring (TDM) in non–critically ill adults hospitalized from March 1, 2017-December 31, 2021. Patients were included if they received intravenous vancomycin for ≥72 hours with a documented trough target of 10 to 15 mg/L, had ≥1 serum vancomycin trough concentration assessed at steady state, and received ≥2 doses of vancomycin after serum assessment with or without dose adjustment. Using an online vancomycin calculator with Bayesian modeling, the study team input patients’ trough-based dosing data to retrospectively estimate the initial vancomycin AUC exposure achieved and the initial and post-trough TDD recommended for an AUC target of 400 to 600 mg⸱h/L. The initial and post-trough vancomycin total daily dose (TDD) derived by the online calculator were compared with the actual doses used for each patient when a trough concentration of 10 to 15 mg/L was targeted.

**Figure:**
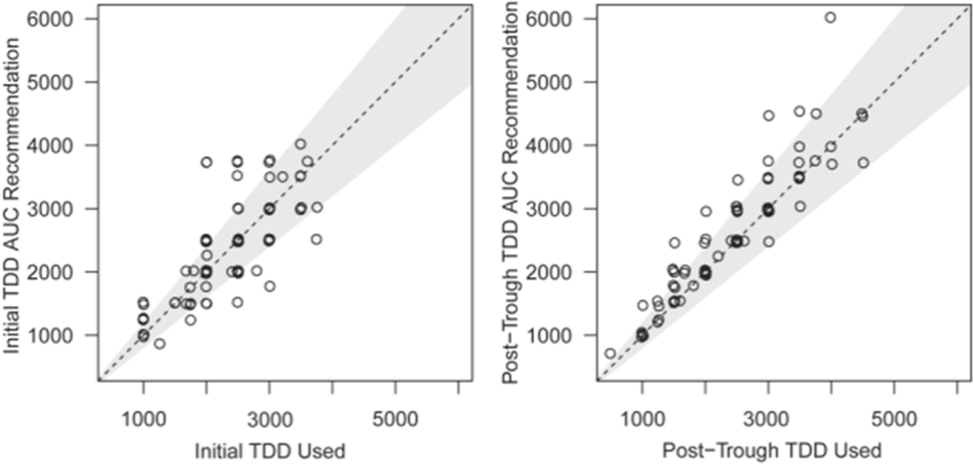
Comparison of Trough-Based and AUC-Based Dosing of Vancomycin

AUC indicates area under the concentration–time curve; TDD, total daily dose. Dashed line indicates where the 2 methods are equal; shading indicates where the 2 methods are within 20% of each other

**Results:**

In total, 89 patients were included in the study. The actual vancomycin TDD with trough monitoring (target, 10-15 mg/L) was within 20% of the recommended AUC-based TDD for 62 patients (70%) with initial dosing and for 74 (83%) after the initial trough concentration. Nephrotoxicity was rarely observed (2%) and the median duration of vancomycin was 4 days.

**Conclusion:**

Given the frequency of dosing similarity between 400-600 mg·h/L AUC and 10-15 mg/L trough-based dosing, vancomycin trough-based monitoring to a goal of 10-15 mg/L is an option warranting further exploration.

**Disclosures:**

All Authors: No reported disclosures

